# Exploring the links between dispositions, romantic relationships, support networks and community inclusion in men and women

**DOI:** 10.1371/journal.pone.0216210

**Published:** 2019-05-07

**Authors:** Eiluned Pearce, Rafael Wlodarski, Anna Machin, Robin I. M. Dunbar

**Affiliations:** Social & Evolutionary Neuroscience Research Group, Department of Experimental Psychology, University of Oxford, Oxford, United Kingdom; Northwestern University, UNITED STATES

## Abstract

Most studies of social cognition have focused on dyadic relationships, and rather few have looked at how we engage with individuals in the wider social world into which we are embedded. Here we use principle component analysis (PCA) and path analysis to explore how different aspects of human sociality interact. We demonstrate two distinct clusters in both sexes relating to (i) romantic relationships and (ii) wider social engagement, such as that with the local community. These two domains of relationship were associated with different dispositional traits: individual variation in impulsivity in the former, and in empathy and avoidant attachment in the latter. Although these clusters were broadly similar across both sexes, clearer differentiation is evident in males. In females only, support network size was positively related to the anxious dimension of attachment and, unlike in males, was not related to feelings of inclusion in the local community. This suggests that support networks may play different roles in the two sexes, indicating a productive line of future research. These findings have important practical applications: loneliness interventions that target the specific type of relationship that is felt to be lacking and the associated dispositional traits are likely to be more effective than more generic approaches.

## Introduction

The social world is by far the most complex aspect of our environment. In part, its challenge arises from the fact that it is dynamic and subject to unpredictable and continuous change over time, as the individuals that make it up fall in and out of favour with each other. Handling this complexity requires cognitive skills, such as empathy and mentalising, that are not required for other non-social tasks. These skills are cognitively demanding in terms of both information processing and neural recruitment [1–7]. Our ability to navigate successfully through this social world also depends crucially on the ability to inhibit prepotent responses: an inadvertent comment or an injudicious social interaction can easily destabilise not only our own dyadic relationships with others, but also relationships between third parties in one’s social network [8]

Most studies of social relationships and social cognition typically focus on close dyadic relationships (romantic relationships or special friendships) and fail to distinguish between relationships of different quality. In doing so, they ignore the wider social network within which an individual is embedded, and which are now known to have very significant consequences for our health, wellbeing and life satisfaction in addition to more intimate connections [9–22]. That the number and quality of our social relationships can have such dramatic–and unanticipated–effects on our health and wellbeing raises pressing questions both about the kinds of cognitive processes that are involved in building and maintaining social relationships and whether different mechanisms are involved in different types of relationships.

In this paper, we use data collected as part of a large-scale study on the genetics of human sociality [23–25] to explore how attitudes and behavior in relation to different domains of sociality relate to each other in healthy adult males and females. We look at three types of relationship: (i) romantic relationships, (ii) the size of the support network of people whom we would rely on in a crisis (our personal “support clique”), and (iii) feelings of inclusion in the local community. These correspond to different layers of intimacy within an individual’s social network (e.g. [26,27]). We also examine how different aspects of disposition may underlie individual variation in these layers of the social network: we look at the roles of individual differences in (i) empathy (the ability to put oneself in another’s shoes), (ii) attachment style (the degree to which one anxiously fears abandonment or avoids intimacy by keeping others at a distance), and (iii) impulsivity (the tendency to act on a whim without considering the consequences).

## Material and methods

### Participants

Data were collected from healthy adults from the UK population attending three science festivals and a museum. Following [23,25], we focus on White participants without a history of mental illness. Participants were required to identify their ethnicity from a standard list of categories: ‘White British’, ‘White (other)’, ‘Indian’, ‘Pakistani’, ‘White Irish’, ‘Mixed’, ‘Black Caribbean’, ‘Black African’, ‘Bangladeshi’, ‘Chinese’, ‘Other Asian’, ‘Black (other)’, and ‘Other’. Participants in the ‘White British’, ‘White (other)’ and ‘White Irish’ were amalgamated for the purpose of this study. Not all participants completed all the survey questions, so the final sample size comprised 398 females (*M* = 38 years, *range* = 18–74 years) and 324 males (*M* = 43 years, *range* = 18–75 years, *N* = 315). The associated data are available in S1 Table.

The study was approved by the University of Oxford Central University Research Ethics Committee (CUREC Ref: MS-IDREC-C2-2015-005), and participants completed consent forms before taking part.

Previous papers from the same study have been published on the associations between the social measures and specific gene variants in both the White sample with no history of mental illness used in this current paper [23], and in the remaining non-white and sub-clinical samples [24]. In addition, analysis of data from this study exploring the relationships between digit ratio, genetic variants and social measures has also been published [25]. Whereas the previous papers explored genetic associations, in contrast the current paper investigates associations between the different social measures. These interrelationships between the social measures have not been previously reported and here we present new analyses of the social measures data only (that is, excluding the genetics data), in order to explore the relations between these dimensions of sociality independently of examining the underlying biology.

### Procedure

Participants in the study completed a set of questionnaires on mobile devices. Descriptive statistics for all variables are given in S2 Table.

To study disposition, we measured empathy, attachment style and impulsivity. To measure empathy we used the Reading the Mind in the Eyes task (RMET), which was validated on a UK sample consists of 36 photos of the eye region of faces expressing different emotions that are presented to the participant one at a time [28]. The participant is asked to identify the correct emotion being expressed from four options, such as ‘ashamed’, ‘nervous’, suspicious’, and ‘indecisive’. Higher scores indicate greater accuracy of identifying emotions.

In addition, we used the short-form Empathy Quotient (EQ), which was validated in a UK sample [29]. This consists of 22 items, 6 of which are reversed scored, such as ‘I find it difficult to judge if something is rude or polite’ (reversed scored) and ‘I can easily tell if someone wants to enter a conversation’. Participants rate the extent to which they agree or disagree with each statement on a 4-point scale anchored as ‘strongly disagree’, slightly disagree’, slightly agree’ and ‘strongly agree’. Means scores are taken to account for missing responses and higher scores indicate greater empathy. Whereas the RMET scale had a relatively low reliability (Cronbach’s α = 0.585), EQ had a high reliability (Cronbach’s α = 0.902).

To measure the Anxious and Avoidant dimensions of attachment style we modified the short-form Experiences of Close Relationships scale (ECR) to relate to ‘close friendships’ rather than romantic relationships [30]. This measure comprises 12 items (4 reverse scored), 6 for each of the two dimensions. For each item participants provide a score on a 7-point scale from ‘strongly disagree’ to ‘strongly agree’ for statements such as ‘I am nervous when close friends get too close to me’ (avoidant) and ‘My desire to be very close sometimes scares people away’ (anxious). Higher scores indicate higher levels of Anxious or Avoidance attachment. We analysed these as two continuous dimensions of attachment rather than categorising participants into different attachment styles, since categorisation means a loss of power and precision (see [31] for the original ECR measure and discussion). In addition, we used the short-form Barratt Impulsiveness Scale [32] as a self-report measure of Impulsivity, which comprises 15 items (6 reverse-scored) rated on a 4-point scale anchored at ‘Rarely/Never’, ‘Occasionally’, ‘Often’ and ‘Almost always/always’, such as ‘I act on the spur of the moment’ and ‘I save regularly’ (reversed scored). Higher scores indicate greater impulsivity. Both attachment subscales had relatively high reliability (anxious Cronbach’s α = 0.735, avoidant Cronbach’s α = 0.777), as did the Impulsivity scale (Cronbach’s α = 0.795).

To measure attitudes and behavior in relation to sexual relationships, we measured sociosexual orientation, using the revised Sociosexual Orientation Inventory (SOI) [33]. This comprises three sections, with a total of 9 items (three in each section), with higher combined scores indicating that an individual is more promiscuous and willing to participate in short-term sexual relationships. The first section asks how many sexual partners an individual has had (i) only once, (ii) without being in a committed relationship, and (iii) in the past 12 months. Participants are required to pick one of 9 categories demarcated as single values for 0 through 4, then as combined categories ‘5–6’, ‘7–9’, ‘10–19’ and ‘20 or more’. The second section comprises three statements, such as ‘Sex without love is OK’, for which the participant is asked to rate the extent of their agreement on a 9-point scale anchored at ‘strongly disagree’, ‘neutral’ and ‘strongly agree’. One of these statements, ‘I do not want to have sex with a person until I am sure that we will have a long-term, serious relationship’, is reversed-scored. The final section asks participants to rate three questions regarding the frequency of fantasies and sexual arousal on a 9-point scale ranging from ‘never’ to ‘at least once a day’.

If the participant was in a sexual relationship, we also used the Relationship Assessment Scale (RAS) [34,35] to assess relationship satisfaction, with higher scores indicating greater satisfaction. The original measure is made up of 7 questions, 2 of which are reverse scored, which participants are asked to answer on a 3-point scale with anchors that vary between items. For example, ‘how well does your partner meet your needs?’ is answered from a choice of ‘poorly’, ‘average’ or ‘extremely well’. However, to maintain consistency with the form of the other measures used in the survey, we rephrased each question as a statement, such as ‘my partner meets all my needs’ that was answered on a 5-point scale ranging from ‘strongly’ disagree’ to ‘strongly agree’. Both the SOI and RAS scales had high reliability (Cronbach’s α = 0.850 and 0.910 respectively).

To explore social relationships in the wider social network, we measured participants’ Support Social Network Size by asking them to record their relationship to the individuals they would turn to for help and support during times of difficulty and distress, and totalling the number of individuals listed ([7,36] following [37,38]). In addition, we measured how integrated or close participants felt to their local community using a modified version of the Inclusion of Other in Self scale, using the label ‘community’ rather than ‘other’ [39]. This measure consists of a sequence of seven diagrams, each of which comprises two circles, which become increasingly overlapped as the scale moves from 1 to 7. The labels of these two circles were modified to ‘self’ versus ‘community’ (rather than ‘other’).

### Analyses

Descriptive statistics are given in S2 Table separately for females and males. We conducted principle component analyses (PCA) in IBM SPSS Statistics for mac, Version 24 (IBM Corp., Armonk, NY, USA). We excluded RAS scores to maximize sample size: 135 females and 95 males did not report being in a romantic relationship at the time of the survey and so did not complete the RAS. We also excluded RMET because initial examination of the correlation matrix indicated very low correlations between this variable and the others (none were above *r* = 0.25). Data for the remaining seven variables yielded sample sizes of 398 females and 324 males. A sample of 300 is generally considered acceptable for PCA [40]. KMO scores were >0.6 and Bartlett’s tests were significant (p<0.0001) for both males and females. Oblique (direct oblimin) rotation of the factors was used. Initially factors with eigenvalues >1 were extracted but examination of scree plots led to forced extraction of three factors (see Results).

To examine the structural relationships between the variables, we conducted path analyses. We ran multiple linear regressions predicting each variable in turn from all the remaining variables, and used the standardized ß values to identify significant relationships between pairs of variables. It should be noted that all these relationships were reciprocal. To explore potential sex differences in how different facets of sociality interlink, we conducted these analyses separately for male and female participants (see S3 & S4 Tables for the partial relationships between the variables from multiple linear regressions in females and males respectively).

## Results

### PCA factors

#### Females

Based on eigenvalues greater than 1, two factors were extracted for females, which together explained 47% of the variance. The first factor included a negative loading of avoidant attachment (-0.760), and positive loadings of EQ (0.718), IOS (0.639) and network size (0.492). The second factor showed positive loadings of impulsivity (0.733), anxious attachment (0.613) and SOI (0.569). However, a scree plot suggested that three factors could be extracted and together these explained 61% of the variance (Table 1). For females the first factor included EQ, avoidant attachment and IOS, whereas the second factor included impulsivity and SOI. The third factor had positive loadings for anxious attachment and network size, but both these variables also loaded, albeit to a lesser extent, onto the first factor. Given that a loading below 0.4 is not considered meaningful [40], the loading of anxious attachment in particular on Factor 1 should probably be ignored.

**Table 1:** PCA factor weightings for females and males. The strongest loadings for each variable are highlighted in grey.

	Variables	Factor 1	Factor 2	Factor 3
**Females**	EQ^a^	.753		
	IOS^b^	.623		
	Avoidant^c^	-.733		
	Network size	.454		.694
	Anxious^c^	-.337		.763
	Impulsivity		.807	
	SOI^d^		.727	
**Males**	EQ^a^	.527		
	IOS^b^	.663		
	Avoidant^c^	-.786		
	Network size	.667	-.380	
	Anxious^c^			.898
	Impulsivity		.661	.494
	SOI^d^		.840	

^a^EQ: Empathy Quotient

^b^IOS: Inclusion of Other in Self scale

^c^Avoidant/Anxious: scores on dimensions of Attachment measured by the Experience in Close Relationships scale

^d^SOI: Sociosexual Orientation Inventory.

#### Males

In males, two factors with eigenvalues >1 were extracted, accounting for 47% of the variance. The first had a negative loading for avoidant attachment (-0.765), and positive loadings for IOS (0.686), EQ (0.589) and network size (0.569). The second factor had positive loadings for impulsivity (0.835), SOI (0.658) and anxious attachment (0.511). The two factors were completely differentiated. However, a scree plot suggested that three factors could be extracted and together these explained 61% of the variance (Table 1). The first factor involved avoidant attachment, network size, IOS and EQ. The second factor comprised SOI and impulsivity, while the third factor included just anxious attachment. However, impulsivity also loaded onto the third factor, and network size also loaded negatively onto the second factor (Table 1). For males support network size was most strongly loaded onto Factor 1 along with IOS, with a weaker loading onto Factor 2 along with SOI, although the latter is below the recommended cut-off of 0.4 [40].

### Path analysis

**Fig 1:**
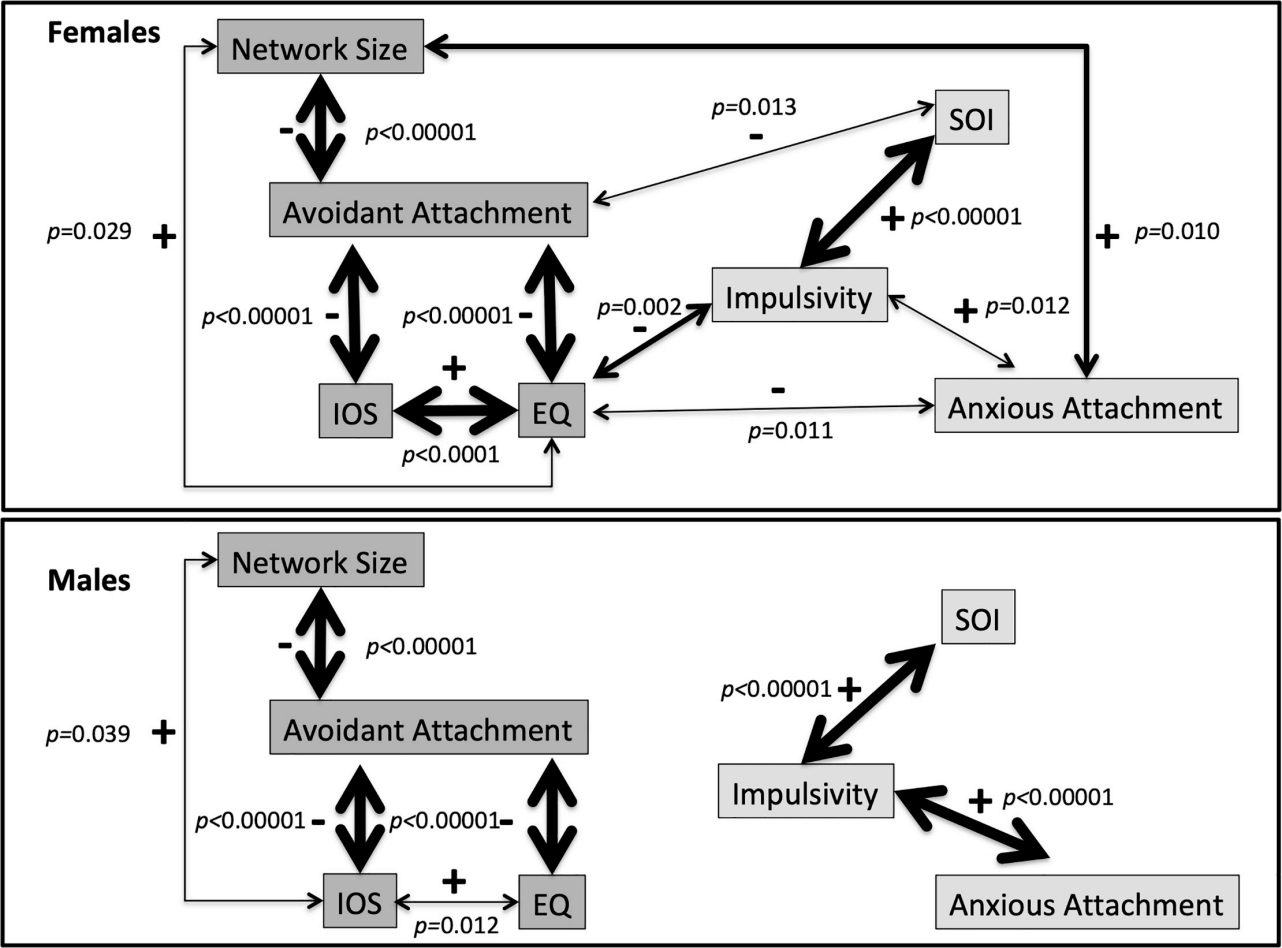
**Path analyses between dispositional and social variables in females (top) and males (bottom).** The line weights of the arrows represent p-values of *p*<0.05, *p*<0.01 and *p*<0.001: the thicker the line the lower the p-value. The two PCA clusters are shown in different shades of grey. EQ: Empathy Quotient; IOS: Inclusion of Other in Self scale; SOI: Sociosexual Orientation Inventory; Avoidant/Anxious: scores on dimensions of Attachment measured by the Experience in Close Relationships scale.

Fig 1 shows the path analyses for females and males. In males, there is a particularly clear distinction between sociosexual orientation, impulsivity and anxious attachment on the one hand and, on the other, engagement in the wider social network (support network size and feelings of inclusion in the local community), empathy and avoidant attachment. Although these two clusters are also evident for females, the path analysis suggests that these clusters are more integrated in females. The links between the two clusters in females are via avoidant attachment and empathy. Moreover, in females, but not males, network size shows a significant positive relationship with anxious attachment. In contrast, in males, support network size is significantly positively associated with IOS, but this association was not found for females.

## Discussion

Overall, the results indicate a distinction between romantic relationships and non-sexual relationships. The first two factors extracted through PCA are broadly similar in males and females, aligning with engagement with wider social networks (Factor 1) as opposed to romantic relationships (Factor 2). Each of these factors includes associated dispositional traits: degree of empathy and avoidant attachment in the case of wider social engagement (Factor 1), and impulsivity with respect to sociosexual orientation (Factor 2). Anxious attachment loaded onto a third factor in both sexes. If only the strongest loadings are considered, this suggests three factors that can be broadly delineated as (i) wider social engagement, (ii) romantic relationships and (iii) the anxious dimensions of attachment.

The wider social engagement (Factor 1) and romantic relationship (Factor 2) clusters found for both sexes in the PCA were confirmed in the path analysis, and common pathways between the sexes are in consistent directions. However, there are also important differences between the sexes. In males, there was a very clear distinction between, on the one hand, sociosexual orientation, impulsivity and anxious attachment and, on the other hand, engagement with broader networks, both in terms of supportive close relationships and feelings of inclusion in the wider community, and the associated dispositional traits of the avoidant dimension of attachment and empathy. These clusters echo the two factors extracted using the eigenvalue >1 criterion, and reflect Factor 1 and a cluster combining Factors 2 and 3.

In contrast, although these two clusters comprising wider social engagement and romantic relationships are evident in the path analysis for females, there is interconnection between them, suggesting that female social worlds are more integrated than those maintained by males. In addition, it is worth noting that these clusters interconnect through impulsivity and empathy, which are antagonistic. Although there are weaker correlations between SOI and avoidant attachment, and between anxious attachment and EQ, based on the stronger correlation between EQ and impulsivity the path analysis suggests that this antagonistic empathy-impulsivity relationship is the key one in connecting these two social domains for females. This may suggest that the more a female can understand the possible consequences of her actions for others, the less likely she is to behave without pause for thought. The capacity to empathise also appears to play a stronger role in how integrated into her local community a female feels compared to males (Fig 1).

Although impulsivity is significantly correlated with SOI and anxious attachment in both sexes, the relationship between impulsivity and anxious attachment was weaker in females. In addition, it is worth noting that SOI is not linked to anxious attachment in either sex (although in males there was a relatively weak loading from impulsivity onto Factor 3 along with anxious attachment: Table 1). This open triad of correlations might suggest that the anxious attachment dimension to some extent drives impulsivity, which independently translates into sociosexual orientation and behavior. However, the correlational nature of path analysis makes this suggestion tentative and the two-way partial relationships suggest that feedback between these variables is also likely.

Another point to note is that network size and feelings of inclusion into the local community are only significantly positively related in males (Fig 1). In contrast, in females support network size is significantly linked to self-reported empathy scores, and there is a stronger relationship between empathy and feelings of inclusion in the local community compared to males. This may reflect the possibility that women maintain the different layers of their social network independently of each other, whereas men may access broader communities comprising acquaintances and weaker ties through their intimate support layer. There is some evidence to suggest that males are more focused on collective bonding in groups and females more focused on relational bonding and one-on-one interactions [41–44]. If male support networks were also part of their local community, this might mean that the larger their network the more comfortable and integrated the men felt in that community, since males seem to be more comfortable in groups than in dyads (discussed in [41]). Female close relationships tend to be more intimate than male friendships [45,46], but the maintenance of such intimacy requires disproportionate time investment [27]. Consequently, it is unlikely that females can create and maintain relationships in their outer layers in the same way as their inner layers, and this may be reflected in the apparent divergence between female support networks and community integration found here.

This divergence between focus on collective or relational belonging between the sexes might also explain why the male social world seems to be more delineated between romantic relationships (relational bonding) and wider networks (collective bonding), whereas females may see both in terms of relational bonding and so the two domains are less differentiated. Alternatively it may be the case that male participants could more easily conceptualise their local community as a collective entity [44], and therefore rate their feelings of closeness with that collective as a group, whereas females may conceptualise their community as comprising individual relationships and therefore found it more difficult to give a combined rating.

Interestingly, in females support network size seems more closely aligned with anxious attachment than with feelings of inclusion in the local community and empathy. It may be that, given the importance of close friendships for females, women who fear abandonment (i.e. have highly anxious attachment styles) are more likely to build redundancy into their networks: a pool of close associates from which to choose in case a particular individual defects. This may be one explanation for why women consistently have larger support cliques than men, in most studies significantly so [7,37,47,48]. It may also reflect the fact that women seem to have more intense close social relationships than men do [49]. Having a larger social support network might cumulatively increase anxiety around each these intense relationships ending.

Together, these results indicate two separate but linked social domains in both sexes: romantic relationships and wider social networks, which are each linked with different dispositional factors: impulsivity in the case of the former and avoidant attachment and empathy with respect to the latter. Whereas males seem to operate as if these domains are separate, at least regarding the measures used here, females seem to have more integrated social cognition systems in that the underlying dispositions seem to interact to a greater extent. It is possible that this reflects a sex difference in genomic imprinting: in mammals, neocortex volume is inherited through female line genes, whereas the limbic system is inherited through male line genes [50], and this may have implications for social style as well as sociosexual behaviour [51,52].

### Strengths and limitations

This study builds on past work by looking at different facets of the social world and associated individual dispositional characteristics in parallel, in order to look at the interplay between them, rather than narrowly focusing on one or two at time. The findings have important implications for understanding individual differences in dispositional traits and how they translate into social behaviour, as well as practical applications in terms of interventions for loneliness. Feeling disconnected can happen both with respect to romantic relationships and to the wider social network, and our results suggest that interventions need to specifically target one or the other because they are relatively distinct domains. However, more generic interventions might be more successful in women, because changes may be more likely to ripple between these different types of relationship if common underlying dispositional traits, such as the ability to empathise, are targeted.

Although this study brings greater insight into how different facets of human sociality and individual dispositions interact, future work should aim to incorporate more objective measures rather than self-report, as well as a greater number of different dimensions, such as relationship quality and other types of relationships such as with kin and colleagues, in order to more fully decipher the complex interplay of human social worlds. Moreover, the sample was limited to UK-based participants who reported being ethnically White and no history of mental illness, so these findings cannot be generalised to other demographic samples. For instance, there may be important cultural differences in how males and females manage their social worlds. In addition, there may have been confounding variables that we did not take into account when asking people about their current social worlds, such as significant life events.

## Supporting information

S1 Table Data file. Data for 'Exploring the links between dispositions, romantic relationships, support networks and community inclusion in men and women'.
(XLSX)

S2 Table Descriptive statistics of variables included. (PDF)

S3 Table Path analysis regressions for females. Partial relationships used to conduct the path analysis for females, controlling for all other variables in a multiple linear regression. ns = not significant. R^2^ values are given for the full models predicting each variable from all other variables.
(PDF)

S4 Table Path analysis regressions for males. Partial relationships used to conduct the path analysis for males, controlling for all other variables in a multiple linear regression. ns = not significant. R^2^ values are given for the full models predicting each variable from all other variables.
(PDF)
