# Supplementary material for: Exploring the links between dispositions, romantic relationships, support networks and community inclusion in men and women
Source: PLoS One. 2019 May 7;14(5):e0216210. doi: 10.1371/journal.pone.0216210 (PMC6504087; doi:10.1371/journal.pone.0216210)
Supplement: S2 Table — (PDF) [file pone.0216210.s002.pdf]

<u>**S2 Table**</u>

'Exploring the links between dispositions, romantic relationships, support networks and community inclusion in men and women'

Eiluned Pearce, Rafael Wlodarski, Anna Machin & Robin I. M. Dunbar

Table S2. Descriptive statistics of variables included.

		N	Minimum	Maximum	Mean	SD
Female	Age	392	18	74	38.47	15.95
	EQ	398	1.32	4.00	3.15	.45
	IOS	398	1	7	3.73	1.44
	Anxious Attachment	398	1.00	6.33	3.10	1.19
	Avoidant Attachment	398	1.00	6.33	2.80	1.15
	Impulsivity	398	1.00	3.40	1.97	.43
	SOI	398	1.00	8.33	3.39	1.49
	Support Network Size	398	1	19	8.19	3.98
Male	Age	315	18	75	43.13	17.24
	EQ	324	1.59	3.82	2.85	.44
	IOS	324	1	7	3.40	1.50
	Anxious Attachment	324	1.00	6.33	3.07	1.08
	Avoidant Attachment	324	1.00	6.50	3.45	1.16
	Impulsivity	324	1.00	3.27	1.98	.40
	SOI	324	1.13	9.00	4.34	1.56
	Support Network Size	324	0	19	6.89	3.79
