# Supplementary material for: Exploring the links between dispositions, romantic relationships, support networks and community inclusion in men and women
Source: PLoS One. 2019 May 7;14(5):e0216210. doi: 10.1371/journal.pone.0216210 (PMC6504087; doi:10.1371/journal.pone.0216210)
Supplement: S3 Table — Partial relationships used to conduct the path analysis for females, controlling for all other variables in a multiple linear regression. ns = not significant. R2 values are given for the full models predicting each variable from all other variables. (PDF) [file pone.0216210.s003.pdf]

<u>**S3 Table**</u>

‘Exploring the links between dispositions, romantic relationships, support networks and community inclusion in men and women’

Eiluned Pearce, Rafael Wlodarski, Anna Machin & Robin I. M. Dunbar

Table S3. Path analysis regressions for females. Partial relationships used to conduct the path analysis for females, controlling for all other variables in a multiple linear regression. ns= not significant. R^2 values are given for the full models predicting each variable from all other variables.

	**EQ**	**IOS**	**Avoidant Attach.**	**Support Network Size**	**Anxious Attach.**	**Impulsivity**
**EQ** R^2=0.21						
**IOS** R^2=0.14	t_{397}=3.54, p<0.0001					
**Avoidant Attachment** R^2=0.25	t_{397}= -5.67, p<0.0001	t_{397}= -4.10, p<0.0001				
**Support Network Size** R^2=0.11	t_{397}= 2.19, p=0.029	ns	t_{397}= -3.96, p<0.0001			
**Anxious Attachment** R^2=0.07	t_{397}= -2.56, p=0.011	ns	ns	t_{397}=2.59, p=0.010		
**Impulsivity** R^2=0.11	t_{397}= -3.07, p=0.002	ns	ns	ns	t_{397}= 2.54, p=0.012	
**SOI** R^2=0.10	ns	ns	t_{397}= -2.51, p=0.013	ns	ns	t_{397}=4.59, p<0.0001
