# Supplementary material for: Exploring the links between dispositions, romantic relationships, support networks and community inclusion in men and women
Source: PLoS One. 2019 May 7;14(5):e0216210. doi: 10.1371/journal.pone.0216210 (PMC6504087; doi:10.1371/journal.pone.0216210)
Supplement: S4 Table — Partial relationships used to conduct the path analysis for males, controlling for all other variables in a multiple linear regression. ns = not significant. R2 values are given for the full models predicting each variable from all other variables. (PDF) [file pone.0216210.s004.pdf]

S4 Table

‘Exploring the links between dispositions, romantic relationships, support networks and community inclusion in men and women’

Eiluned Pearce, Rafael Wlodarski, Anna Machin & Robin I. M. Dunbar

Table S4. Path analysis regressions for males. Partial relationships used to conduct the path analysis for males, controlling for all other variables in a multiple linear regression. ns= not significant. R^2 values are given for the full models predicting each variable from all other variables.

	**EQ**	**IOS**	**Avoidant Attach.**	**Support Network Size**	**Anxious Attach.**	**Impulsivity**
**EQ** R^2=0.13						
**IOS** R^2=0.16	t_{323}= -2.53, p=0.012					
**Avoidant Attachment** R^2=0.24	t_{323}= -4.33, p<0.0001	t_{323}= -4.53, p<0.0001				
**Support Network Size** R^2=0.13	ns	t_{323}=2.10, p=0.039	t_{323}= -4.99, p<0.0001			
**Anxious Attachment** R^2=0.08	ns	ns	ns	ns		
**Impulsivity** R^2=0.20	ns	ns	ns	ns	t_{323}=4.94, p<0.0001	
**SOI** R^2=0.15	ns	ns	ns	ns	ns	t_{323}=6.59, p<0.0001
